# Does ongoing antithrombotic therapy increase the risk of revision after trochanteric fracture fixation? A retrospective cohort study with competing risk analyses

**DOI:** 10.1007/s00402-025-06121-2

**Published:** 2025-11-07

**Authors:** Roberta Laggner, Florian Bur, Michael Humenberger, Martin Frossard, Stefan Hajdu, Valerie Weihs

**Affiliations:** Department of Orthopedics and Trauma Surgery,, Medical University of Vienna, Vienna, Austria

**Keywords:** Trochanteric femoral fracture, Antithrombotic therapy, Cephalomedullary nailing, Revision surgery, Infection, Mortality

## Abstract

**Background:**

Trochanteric femoral fractures are associated with high morbidity and mortality with a substantial proportion of patients presenting with ongoing antithrombotic therapy (ATT). Evidence regarding the impact of ATT on surgical outcomes and complication rates in this population remains limited. The purpose of this study was to evaluate revision rates, infection risk, surgical timing, and mortality in patients with trochanteric fractures receiving ATT.

**Methods:**

We retrospectively analyzed 656 patients who underwent cephalomedullary nailing for trochanteric femoral fractures between January 2021 and December 2024. Patients were stratified by pre-injury ATT status. The primary outcome was revision surgery; secondary outcomes included infection requiring revision, surgical timing, and mortality at predefined intervals.

**Results:**

Of 656 patients, 319 (48.6%) presented with pre-injury ATT. Revision surgery was required in 33 patients (5.0%) and did not differ significantly between ATT and non-ATT groups (6.0% vs. 4.2%, *p* = 0.291). Infections occurred in 1.2% of patients, with no excess risk in ATT patients. Patients with pre-injury ATT had significantly higher mortality rates (*p* = 0.005), although the one-year mortality did not differ significantly (23.8% vs. 23.1%, *p* = 0.989) between the two groups. Competing risk analyses revealed a significant impact of pre-injury ATT on the mortality (*p* = 0.004) but not on the revision rates (*p* = 0.311).

**Conclusions:**

In this large cohort, nearly half of all patients with trochanteric fractures were on ATT at admission. ATT was not associated with increased revision or infection risk. While overall mortality was higher in patients receiving ATT, one-year mortality was comparable between groups, indicating that early excess risk is more likely related to age and comorbidity. These findings suggest that ATT itself does not predispose to adverse surgical outcomes.

## Introduction

Trochanteric femoral fractures are among the most common injuries in the elderly population and are associated with significant morbidity, mortality, and socioeconomic burden [1, 2]. With the ongoing demographic shift and increasing life expectancy, the incidence of these fractures is expected to rise steadily. Surgical fixation using an intramedullary proximal femoral nail has become the gold standard in treating trochanteric fractures due to its biomechanical advantages and the potential for early mobilization [3].

Despite standardized surgical techniques and modern implant designs, postoperative complications remain a relevant issue. These complications can include mechanical failure, infection, non-union, malunion, and cut-out or cut-through phenomena, often necessitating revision surgery [3–5]. Reported revision rates vary depending on fracture complexity, implant type, and patient characteristics, but are consistently associated with prolonged recovery, increased healthcare costs, and worse functional outcomes. Understanding the frequency and determinants of such complications is crucial for guiding clinical decision-making and optimizing patient care [5–9].

An increasing proportion of hip fracture patients are admitted under antithrombotic therapy (ATT), including vitamin K antagonists, direct oral anticoagulants (DOACs), and antiplatelet agents. Prior studies estimate that 15–40% of patients present with ATT at the time of hip fracture [10, 11], reflecting both the aging population and the high prevalence of cardiovascular comorbidities in this group. The presence of ATT at admission raises concerns about perioperative bleeding risk, need for transfusion, and potential predisposition to hematoma-related infection. At the same time, surgical delay—often introduced for drug washout or bridging therapy—is a well-recognized risk factor for increased mortality and complications [12–16].

Recent evidence suggests that early surgery, even under active anticoagulation, may be safe [17]. A prospective matched-pair study reported that fixation of proximal femoral fractures within 24 h in patients on DOAC therapy did not increase perioperative blood loss, transfusion requirements, or complication rates, while significantly reducing the length of stay [18]. These findings challenge the traditional practice of delaying surgery and support the concept that timely surgical fixation should be prioritized even in anticoagulated patients [14].

However, despite these insights, there is limited evidence regarding the specific impact of ATT on revision rates, mechanical failure, and septic complications after trochanteric fracture fixation. This knowledge gap is clinically important, as revision surgery represents a major determinant of morbidity, and infections in particular carry devastating consequences in the elderly.

The aim of this study was therefore to determine the peri- and postoperative complication rates of patients undergoing cephalomedullary fixation of trochanteric femoral fractures, with a specific focus on the influence of pre-injury antithrombotic therapy. Secondary objectives included evaluating surgical timing, comorbidities, and mortality outcomes in relation to ATT status.

## Methods

We conducted a retrospective cohort study of patients admitted with trochanteric femoral fractures who underwent surgical fixation with a cephalomedullary nail between January 2021 and December 2024.

During the study period, a total of 939 patients with a diagnosis of trochanteric femoral fracture were identified through institutional medical records and surgical databases. Patients were eligible for inclusion if they were aged 50 years or older, underwent surgical fixation with a cephalomedullary nail, and had a minimum follow-up of 12 months. A total of 283 patients were excluded for the following reasons: age under 50 years (n = 18), non-surgical management (n = 6), pathological or non-recent fractures, subtrochanteric or multiple fractures (n = 109), missing essential clinical or operative data (n = 53), or lack of a minimum follow-up of 12 months (n = 97). After application of these criteria, 656 patients were included in the final analysis.

Data were retrieved from electronic medical records, operative reports, anesthesia protocols, and follow-up records. Demographic and baseline variables included sex, age and pre-injury antithrombotic therapy including oral anticoagulants and antiplatelet agents.

The primary outcome of interest was the rate of revision surgery, stratified by pre-injury antithrombotic therapy (ATT). Revision surgery was defined as any reoperation of the proximal femur after index surgery. Revision procedures were further categorized as mechanical failures, like cut-out, implant migration, fracture around the implant or hematoma or septic complications like deep surgical site infection requiring implant removal or exchange.

Patients requiring revision surgery were further analyzed. The fracture type was classified according to the AO/OTA classification system. Perioperative characteristics included pre-injury ATT, time from hospital admission to incision (time to surgery), the American Society of Anesthesiologists (ASA) score, age and sex. The time to surgery was analyzed both as a continuous variable and in categories (≤ 12 h, ≤ 24 h, > 48 h).

### Statistics

All data were pseudonymized and stored securely before analysis. Continuous variables were reported as means with standard deviations or medians with interquartile ranges, whereas categorical variables were presented as frequencies and percentages. Group comparisons were performed using chi-square or Fisher’s exact tests for categorical variables and Student’s t-test or Wilcoxon rank-sum tests for continuous variables. Mortality, including 30-day, 90-day and 1-year mortality was reported. Survival analyses were performed using the Kaplan–Meier method, and differences between groups were assessed with the log-rank test. Prognostic factors for revision surgery and mortality were assessed in univariate Cox regression analyses. A two-sided p-value < 0.05 was considered statistically significant. All analyses were performed using IBM SPSS Statistics version 29.0. Given that the risk of death likely exceeded the risk of revision surgery in this cohort of patients, a competing risk analysis of the cumulative incidence (95% confidence interval [95% CI]) of revision surgery with death as a competing event was performed. The competing risk analysis was performed using the cmprsk package in the statistical software R version 4.5.1 (2025-06-13) [19].

The local institutional review board approved the study protocol before data collection was started and waived the need for individual informed consent. All patient records were anonymized and de-identified before analysis. The study was conducted in accordance with the Declaration of Helsinki, the ICH Harmonized Tripartite Guideline for Good Clinical Practice, and the guidelines of the local Institutional Review Board.

## Results

After exclusions, as listed in the Methods Section, 656 patients were included in the final analysis. Of these, nearly half (48.6%, n = 319) presented with pre-injury ATT. Oral anticoagulation (OAC) was documented in 152 patients (47.6%), ATT in 154 patients (48.3%), and low-molecular-weight heparin in 13 patients (4.1%). The remaining 337 patients (51.4%) had no ATT.

Baseline clinical characteristics are summarized in Table 1.

**Table 1:** Clinical characteristics of all patients

All Patients	ATT n = 319	No ATT n = 337	p value
Age median (IQR)	83 (12)	81 (15)	0.003
Revision surgery n (%)	19 (6.0%)	14 (4.2%)	0.291
Time to revision median (IQR)	46 (94)	42.5 (173)	0.872
Overall mortality n (%)	98 (30.7%)	71 (21.1%)	0.005
30-day mortality n (%)	22	11	0.260
90-day mortality n (%)	50	27	0.094
1-year mortality n (%)	76	55	0.989
Time to death median (IQR)	117 (282)	90 (274)	0.158

*ATT* antithrombotic therapy, *IQR* interquartile range

### Characteristics of the revision surgeries

Overall, 33 patients (5.0%) underwent revision surgery after cephalomedullary fixation. The majority of revisions were due to mechanical failure (n = 25, 76%), while eight patients (24%) required revision for septic complications (Table 2).

**Table 2:** Timing of index surgery and type of failure in 33 patients undergoing revision

	Mechanical (n = 25)	Septic (n = 8)	p value
Surgery ≤ 12 h n (%)	13 (52%)	4 (50%)	0.922
Surgery ≤ 24 h n (%)	17 (68%)	5 (62.5%)	0.774
Surgery ≥ 48 h n (%)	3 (12%)	1 (12.5%)	0.970
Type of failure			
Screw failure n (%)	13 (52.0%)		
Hematoma n (%)	4 (16.0%)		
Periimplant fracture n (%)	3 (12.0%)		
Pseudarthrosis n (%)	2 (8.0%)		
Other n (%)	3 (12.0%)		
Acute infection n (%)		5 (62.5%)	
Chronic infection n (%)		3 (37.5%)	

An acute infection was defined as an infection leading to the revision surgery within 6 weeks after the index surgery (see Table 3).

**Table 3:** Characteristics of revision patients according to antithrombotic therapy

Patients with Revision Surgery	ATT n = 19	no ATT n = 14	p value
age median (IQR)	83 (7)	82.5 (11)	0.843
female n (%)	16 (84.2%)	11 (78.6%)	0.678
ASA score 2 n (%)	5 (26.3%	10 (71.4%)	
ASA score 3 n (%)	12 (63.2%)	4 (28.6%)	
ASA score 4 n (%)	2 (10.5%)	0	0.029
Time to surgery median (IQR)	26.3 (37.2)	7.9 (8.1)	0.002
Time to surgery ≤ 12 h n (%)	6 (31.6%)	11 (78.6%)	0.008
Time to surgery ≤ 24 h n (%)	9 (47.4%)	13 (92.9%)	0.006
Time to surgery ≤ 48 h n (%)	15 (78.9%)	14 (100%)	0.067
Type of failure			
Septic n (%)	4 (21.1%)	4 (28.6%)	
Mechanical n (%)	15 (78.9%)	10 (71.4%)	0.618
Time to 1st revision median (IQR)	45 (93)	42.5 (172)	0.957
Death after revision	5 (26.3%	4 (28.6%)	0.886

*ATT* antithrombotic therapy, *IQR* interquartile range, *ASA* score

### Survival analyses

**Fig. 1:**
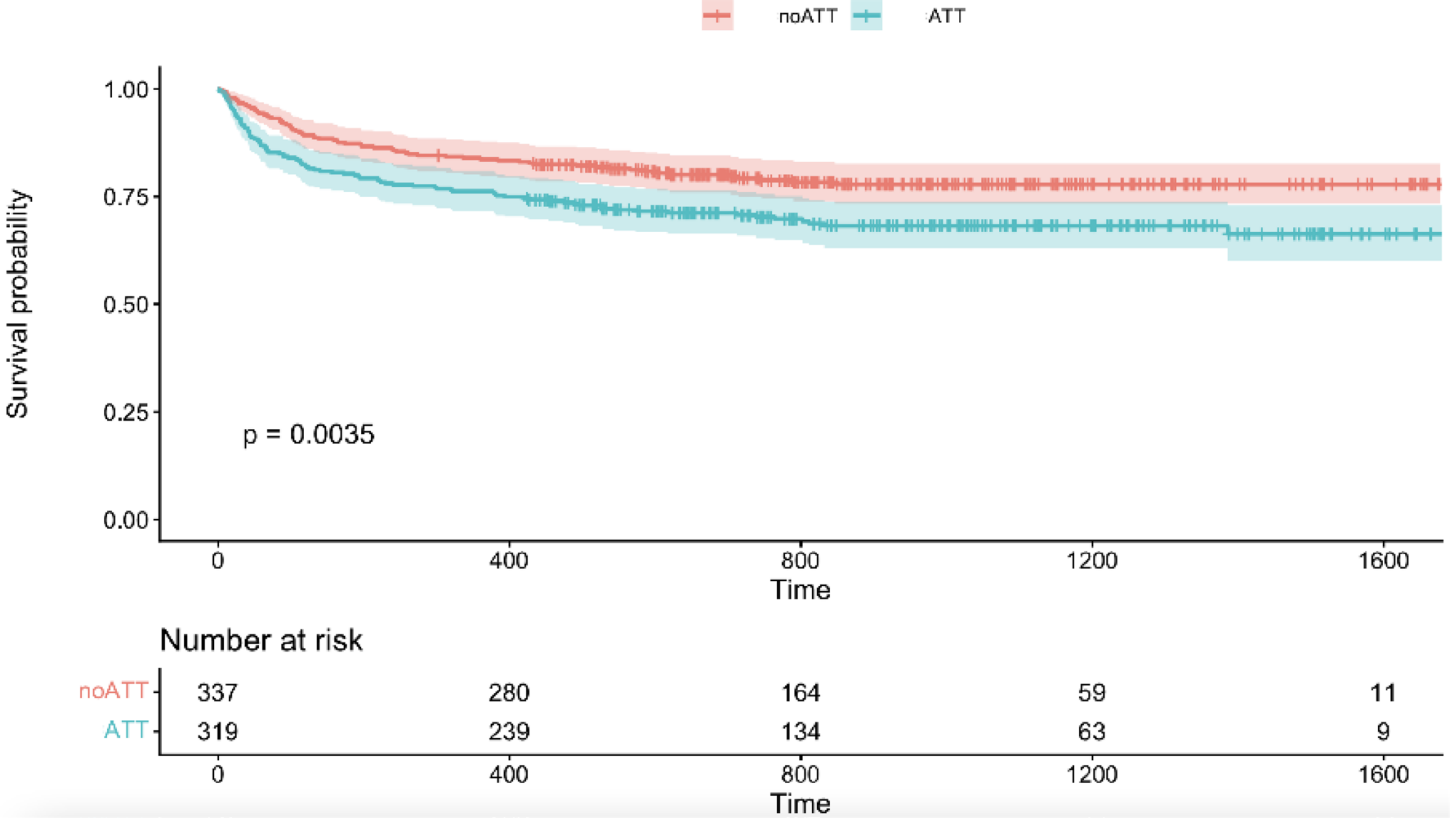
Kaplan–Meier survival curves for patients with and without pre-injury ATT

During follow-up, 169 patients (25.8%) died. Out of them, 131 patients died within the first year after surgery: 33 patients within 30 days, 77 patients within 90 days and 21 patients after 90 days within 1 year after surgery. The remaining 38 patients died after the first year during the follow-up period. The overall mortality rate was significantly higher in patients with ATT compared to those without (30.7% vs. 21.1%, p = 0.005). However, the one-year mortality did not differ significantly between groups (23.8% vs. 23.1%, p = 0.989). Survival analysis revealed a significant influence of pre-injury ATT on the overall survival (Fig. 1). Patients with per-injury ATT had a significantly higher risk of overall mortality than patients without ATT (HR 1.57, 95% CI 1.16–2.12, p = 0.004).

The mortality rate among revision patients was comparable to that of patients without revision (27.3% vs. 25.7%, p = 0.839). After revision surgery, nine patients (27.3%) died during follow-up, without significant differences between ATT and non-ATT subgroups (26.3% vs. 28.6%, p = 0.886).

**Fig. 2:**
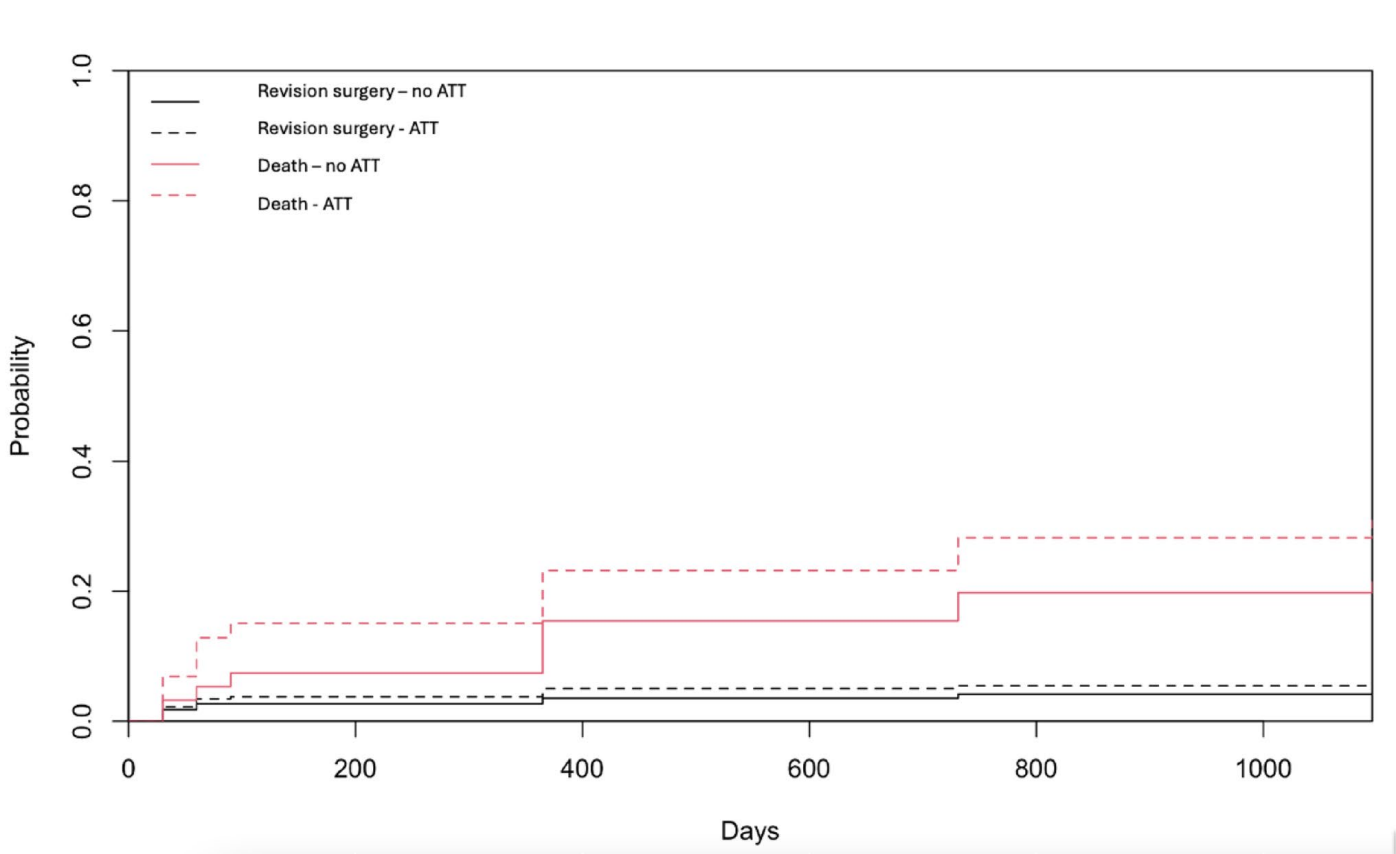
Estimated cumulative incidence curves with revision surgery (black) and death (red) as competing events for patients with and without pre-injury ATT

Competing risk analyses revealed no influence of pre-injury ATT on the revision rate (p = 0.3113) but a significant influence of pre-injury ATT on the survival (p = 0.0043) (Fig. 2).

## Discussion

In this large retrospective cohort, nearly half of all patients presenting with trochanteric fractures were on antithrombotic therapy at admission. The key finding of our analysis is that pre-injury ATT was not associated with an increased risk of revision surgery, whether due to mechanical or septic complications. Despite this finding, our study suggests that pre-injury ATT was not associated with increased hematoma formation or septic complications leading to revision surgery. To our knowledge, this is the first study to specifically investigate revision and infection rates in patients with trochanteric fractures undergoing ATT, thereby adding new evidence to guide perioperative decision-making in this high-risk population.

Nearly half of our trochanteric fracture cohort (48.6%) was on antithrombotic therapy at admission, a higher prevalence than many prior reports, which ranged from approximately 15% to 40%, depending on the patient population and type of antithrombotic agent [10–12]. This elevated prevalence in our study likely reflects the increased age and comorbidity seen in patients with trochanteric fractures. Also, it underscores the importance of understanding ATT-specific outcomes in this high-risk population.

In our cohort, the overall revision rate after cephalomedullary fixation of trochanteric femoral fractures was 5.0%, with the majority due to mechanical failure (76%). This rate is comparable to those reported in the literature: large registry and institutional studies have reported mechanical complication rates ranging from 2 to 7% for intramedullary fixation of trochanteric fractures. However, some series have reported higher rates, depending on the implant type and patient population [5–7]. In our cohort, fixation was predominantly performed using standard cephalomedullary nail systems, including the short Gamma 3 Nail (Stryker) and Proximal Femoral Nail Antirotation (PFNA, Synthes), Trochanteric Femoral Nail Advanced (TFNA Synthes). Reported cut-out rates range from 1.8% as described by Bojan et.al. [7] to 4–5% of cases [20]. Other intramedullary implants, such as Intertrochanteric Antegrade Nail (InterTAN) or Intramedullary Hip Screw (IMHS), have been associated with higher failure rates of up to 12–17% [20].

Given that mechanical failures remain the predominant reason for revision, efforts to optimize implant positioning and bone quality management may be more important than concerns regarding the timing of surgery in patients with antithrombotic therapy. Importantly, we found no evidence that pre-injury antithrombotic therapy had an influence on the type of failure (mechanical vs. septic complication) leading to revision surgery.

Revision due to infection occurred in 8 patients, corresponding to an incidence of 1.2%. This is in line with previously published infection rates following intramedullary fixation of trochanteric fractures, which typically range from 1 to 3% [21]. Notably, no excess of infectious complications was observed among patients with pre-injury ATT, despite theoretical concerns about an increased hematoma-related infection risk in anticoagulated patients. These findings indicate that ATT was not clearly associated with an increased risk of wound-related or implant-related complications in our cohort, but the possibility of subtle effects cannot be excluded. This interpretation aligns with a recent meta-analysis by Wei et al., which compared patients with hip fractures on warfarin versus DOACs and found no significant differences in postoperative infection, thromboembolic events, or revision rates, although hospital stay was longer in warfarin users [22].

While our results are reassuring, they should be interpreted with caution, and further studies are needed to determine whether timely surgical fixation in this patient group can be performed without an increased risk of infection. Although no study has specifically examined infection rates in patients with trochanteric fractures receiving ATT, existing research in broader hip fracture populations suggests that ATT does not substantially increase overall complication rates [23].

Surgical delay is a well-recognized risk factor for worse outcomes, with one study demonstrating that delays greater than 12 h increased the odds of 30-day mortality by 43% (OR 1.43; 95% CI 1.06–1.99; p = 0.025) [24]. Supporting this, a prospective matched-pair analysis found that early surgery (≤ 24 h) in patients on active DOAC therapy did not increase postoperative blood loss or transfusion requirements and was associated with a shorter length of hospital stay [18]. In line with this, Kolodychuk et al. reported that hip fracture patients on DOACs undergoing early surgery (< 48 h) had no increase in transfusion requirements, major bleeding, or 30-day mortality compared with non-anticoagulated patients, further supporting the safety of timely fixation in this setting [17]. Another study observed that DOAC users undergoing early fixation did not experience higher bleeding, wound complications, or mortality compared to controls. However, in this study, surgical delay was modestly longer and 30-day readmission was more common, reflecting their underlying comorbidity burden [16]. In contrast, a recent large multicentre study from the United Kingdom reported that anticoagulated patients underwent surgery on average 7.6 h later than non-anticoagulated patients, with fewer than half (42%) receiving surgery within 36 h, and higher 30-day mortality (HR 1.27, 95% CI 1.03–1.57) [12]. Compared to these findings, the majority of revision patients in our cohort, despite ATT, still underwent surgery within 48 h, and no increase in revision or infection risk was observed. Furthermore, the time-to-surgery did not influence the type of failure (septic or mechanical complications). Taken together, this suggests that institutional ability to ensure timely osteosynthesis may be more relevant to outcomes than ATT itself, and that unnecessary delay for drug washout is not supported by available evidence.

Survival analysis revealed a significant association between ATT and overall mortality, with ATT patients experiencing a 57% higher risk of death during follow-up (HR 1.57, 95% CI 1.16–2.12). This effect was most evident in the early postoperative period, as reflected by the higher overall mortality (30.7% vs. 21.1%), while one-year mortality did not differ significantly between groups. The convergence of survival rates over time suggests that the early disadvantage observed in ATT patients may be attributable to their higher burden of comorbidity and frailty rather than ATT itself. Indeed, patients on ATT in our study were older and had higher ASA classifications, both of which are well-established predictors of mortality in hip fracture populations. Both the Charlson Comorbidity Index (CCI) and ASA scoring systems have been shown to exhibit a strong, stepwise association with one-year mortality in hip fracture patients, with higher categories predicting significantly worse outcomes [25, 26].

Our findings are consistent with these reports and highlight ATT as a marker of frailty rather than an independent driver of poor survival.

Notably, survival did not differ between patients who underwent revision and those who did not, and ATT status had no influence on survival among patients requiring revision. These results reinforce the interpretation that underlying frailty, rather than surgical complications or ATT itself, is the primary driver of survival outcomes. From a clinical standpoint, this emphasizes the importance of careful perioperative optimization and multidisciplinary management of ATT patients, without delaying surgical fixation.

This study has several limitations. Its retrospective design introduces the potential for selection and information bias. The number of septic revisions was relatively small, limiting the power to detect subtle associations with ATT. Furthermore, while patients were stratified by ATT status, we did not differentiate between individual antithrombotic agents or dosing regimens, which may have differing impacts on perioperative risk. Finally, this was a single-center study, which may limit generalizability to other healthcare systems and surgical practices.

## Conclusion

In summary, pre-injury antithrombotic therapy was not associated with higher rates of revision surgery, whether due to mechanical failure or infection, after cephalomedullary nailing of trochanteric fractures. Although overall mortality was higher among ATT patients, this appears to reflect their older age and greater comorbidity burden rather than the use of antithrombotics themselves. Our results indicate that ATT did not increase the risk of revision or infection, supporting that ATT alone should not be considered a contraindication to early osteosynthesis in patients with proximal femur fractures.

## Data Availability

No datasets were generated or analysed during the current study.

## References

[1] Kanis JA, Oden A, McCloskey EV, Johansson H, Wahl DA, Cooper C et al (2012) A systematic review of hip fracture incidence and probability of fracture worldwide. Osteoporos Int 23(9):2239–2256 22419370 10.1007/s00198-012-1964-3 PMC3421108

[2] Feng JN, Zhang CG, Li BH, Zhan SY, Wang SF, Song CL (2024) Global burden of hip fracture: the Global Burden of Disease Study. Osteoporos Int 35(1):41–52 37704919 10.1007/s00198-023-06907-3

[3] Lee C, Kelley B, Gurbani A, Stavrakis AI (2022) Strategies for pertrochanteric fracture reduction and intramedullary nail placement: technical tips and tricks. J Am Acad Orthop Surg 30(18):867–878 36166383 10.5435/JAAOS-D-21-01007

[4] Eardley W, Johansen A (2024) Safety and efficacy in the management of older patients with displaced intracapsular hip fractures. Injury 55(7):111598 38776790 10.1016/j.injury.2024.111598

[5] Lopez-Hualda A, Garcia-Cabrera EM, Lobato-Perez M, Martinez-Martin J, Rossettini G, Leigheb M et al (2024) Mechanical complications of proximal femur fractures treated with intramedullary nailing: a retrospective study. Medicina (Kaunas). 10.3390/medicina60050718 38792901 10.3390/medicina60050718 PMC11123330

[6] Klima ML (2022) Mechanical complications after intramedullary fixation of extracapsular hip fractures. J Am Acad Orthop Surg 30(24):e1550–e1562 36476463 10.5435/JAAOS-D-22-00213

[7] Bojan AJ, Beimel C, Taglang G, Collin D, Ekholm C, Jonsson A (2013) Critical factors in cut-out complication after Gamma nail treatment of proximal femoral fractures. BMC Musculoskelet Disord 14:1 23281775 10.1186/1471-2474-14-1 PMC3543839

[8] Mory N, Saab M, Kaba A, Chantelot C, Jan N (2022) Mortality and functional consequences after revision osteosynthesis for peritrochanteric fractures treated by intramedullary nail: a retrospective study of 312 patients. Orthop Traumatol Surg Res 108(5):103325 35589084 10.1016/j.otsr.2022.103325

[9] Ozdemir E, Okkaoglu MC, Evren AT, Yaradilmis YU, Ates A, Altay M (2021) The cost and consequences of failed osteosynthesis of intertrochanteric femur fractures: a matched cohort study. Indian J Orthop 55(3):629–635 33995866 10.1007/s43465-020-00322-0 PMC8081792

[10] Rostagno C, Cartei A, Polidori G, Civinini R, Ceccofiglio A, Rubbieri G et al (2021) Management of ongoing direct anticoagulant treatment in patients with hip fracture. Sci Rep 11(1):9467 33947928 10.1038/s41598-021-89077-8 PMC8096972

[11] Spitler C, Rutz R, Blackwood N, Wally M, Johnson J, Krause P et al (2025) Management of hip fracture patients on direct oral anticoagulants: a survey of orthopaedic trauma surgeons, systematic review, and meta-analysis. OTA International 8(1):e360 39876981 10.1097/OI9.0000000000000360 PMC11774270

[12] Farhan-Alanie MM, Chinweze R, Walker R, Eardley WGP, collaborators H (2024) The impact of anticoagulant medications on fragility femur fracture care: the hip and femoral fracture anticoagulation surgical timing evaluation (HASTE) study. Injury 55(6):111451 38507942 10.1016/j.injury.2024.111451

[13] Ryan DJ, Yoshihara H, Yoneoka D, Egol KA, Zuckerman JD (2015) Delay in hip fracture surgery: an analysis of patient-specific and hospital-specific risk factors. J Orthop Trauma 29(8):343–348 25714442 10.1097/BOT.0000000000000313

[14] Pincus D, Ravi B, Wasserstein D, Huang A, Paterson JM, Nathens AB et al (2017) Association between wait time and 30-day mortality in adults undergoing hip fracture surgery. JAMA 318(20):1994–2003 29183076 10.1001/jama.2017.17606 PMC5820694

[15] Mullins B, Akehurst H, Slattery D, Chesser T (2018) Should surgery be delayed in patients taking direct oral anticoagulants who suffer a hip fracture? A retrospective, case-controlled observational study at a UK major trauma centre. BMJ Open 8(4):e020625 29705761 10.1136/bmjopen-2017-020625 PMC5931299

[16] Franklin NA, Ali AH, Hurley RK, Mir HR, Beltran MJ (2018) Outcomes of early surgical intervention in geriatric proximal femur fractures among patients receiving direct oral anticoagulation. J Orthop Trauma 32(6):269–273 29432317 10.1097/BOT.0000000000001146

[17] Kolodychuk NL, Godshaw B, Nammour M, Starring H, Mautner J (2023) Early hip fracture surgery is safe for patients on direct oral anticoagulants. OTA Int The Open Access J Orthop Trauma 6(2):e252 10.1097/OI9.0000000000000252 PMC10079331 37034428

[18] Weihs V, Humenberger M, Sturz G, Martin C, Pausch A, Duma A et al (2025) Early surgical fixation of proximal femur fractures under active direct oral anticoagulation (DOAC) therapy does not increase the postoperative blood loss. Results from a prospective cohort study with a matched-pair analysis. Arch Orthop Trauma Surg 145(1):243 40221597 10.1007/s00402-025-05870-4 PMC11993469

[19] Team RC (2025) _R: A Language and Environment for Statistical Computing_

[20] McAleese T, McLeod A, Keogh C, Harty JA (2024) Mechanical outcomes of the TFNA, InterTAN and IMHS intramedullary nailing systems for the fixation of proximal femur fractures. Injury 55(2):111185 38070327 10.1016/j.injury.2023.111185

[21] Halonen LM, Stenroos A, Vasara H, Huotari K, Kosola J (2021) Infections after intramedullary fixation of trochanteric fractures are uncommon and implant removal is not usually needed. Injury 52(6):1511–1516 34057070 10.1016/j.injury.2020.10.076

[22] Wei Y, Chen C, Yu Z, Guo J (2025) Comparing the post-operative complications following surgery for hip fracture of patients who were on warfarin versus patients who were on novel oral anticoagulants: a meta-analysis. Perioper Med (Lond) 14(1):21 39962569 10.1186/s13741-025-00502-2 PMC11834543

[23] Schuetze K, Eickhoff A, Dehner C, Gebhard F, Richter PH (2019) Impact of oral anticoagulation on proximal femur fractures treated within 24 h—a retrospective chart review. Injury 50(11):2040–2044 31543315 10.1016/j.injury.2019.09.011

[24] Warren M, Bretherton C, Parker M (2024) Delay to surgery beyond 12 hours is associated with increased hip fracture mortality. Eur J Orthop Surg Traumatol 34(6):2973–2980 38844565 10.1007/s00590-024-03997-5 PMC11377486

[25] Ek S, Meyer AC, Hedstrom M, Modig K (2022) Comorbidity and the association with 1-year mortality in hip fracture patients: can the ASA score and the Charlson Comorbidity Index be used interchangeably? Aging Clin Exp Res 34(1):129–136 34106421 10.1007/s40520-021-01896-x PMC8795011

[26] Krusic D, Brilej D, Currie C, Komadina R (2016) Audit of geriatric hip fracture care - a Slovenian trauma center analysis. Wien Klin Wochenschr 128(Suppl 7):527–534 27896467 10.1007/s00508-016-1137-z

